# Functional respiratory imaging in relation to classical outcome measures in cystic fibrosis: a cross-sectional study

**DOI:** 10.1186/s12890-021-01622-3

**Published:** 2021-08-04

**Authors:** Eline Lauwers, Annemiek Snoeckx, Kris Ides, Kim Van Hoorenbeeck, Maarten Lanclus, Wilfried De Backer, Jan De Backer, Stijn Verhulst

**Affiliations:** 1 Laboratory of Experimental Medicine and Pediatrics, Faculty of Medicine and Health Sciences, University of Antwerp, Universiteitsplein 1, 2160 Wilrijk, Belgium; 2 Infla-Med Research Consortium of Excellence, University of Antwerp, Antwerp, Belgium; 3 Department of Radiology, Antwerp University Hospital, Edegem, Belgium; 4 Department of Pediatrics, Antwerp University Hospital, Edegem, Belgium; 5 CoSys Research Lab, Faculty of Applied Engineering, University of Antwerp, Antwerp, Belgium; 6 Flanders Make Strategic Research Center, Lommel, Belgium; 7 FLUIDDA NV, Kontich, Belgium; 8 Faculty of Medicine and Health Sciences, University of Antwerp, Antwerp, Belgium

**Keywords:** Cystic fibrosis, Chest computed tomography, Functional respiratory imaging, Quantitative measures

## Abstract

**Background:**

Functional Respiratory Imaging (FRI) combines HRCT scans with computational fluid dynamics to provide objective and quantitative information about lung structure and function. FRI has proven its value in pulmonary diseases such as COPD and asthma, but limited studies have focused on cystic fibrosis (CF). This study aims to investigate the relation of multiple FRI parameters to validated imaging parameters and classical respiratory outcomes in a CF population.

**Methods:**

CF patients aged > 5 years scheduled for a chest CT were recruited in a cross-sectional study. FRI outcomes included regional airway volume, airway wall volume, airway resistance, lobar volume, air trapping and pulmonary blood distribution. Besides FRI, CT scans were independently evaluated by 2 readers using the CF-CT score. Spirometry and the 6-Minute Walk Test (6MWT) were also performed. Statistical tests included linear mixed-effects models, repeated measures correlations, Pearson and Spearman correlations.

**Results:**

39 CT scans of 24 (17M/7F) subjects were analyzed. Patients were 24 ± 9 years old and had a ppFEV_1_ of 71 ± 25% at the time of the first CT. All FRI parameters showed significant low-to-moderate correlations with the total CF-CT score, except for lobar volume. When considering the relation between FRI parameters and similar CF-CT subscores, significant correlations were found between parameters related to airway volume, air trapping and airway wall thickening. Air trapping, lobar volume after normal expiration and pulmonary blood distribution showed significant associations with all spirometric parameters and oxygen saturation at the end of 6MWT. In addition, air trapping was the only parameter related to the distance covered during 6MWT. A subgroup analysis showed considerably higher correlations in patients with mild lung disease (ppFEV_1_ ≥ 70%) compared to patients with moderate to severe lung disease (ppFEV_1_ < 70%) when comparing FRI to CF-CT scores.

**Conclusions:**

Multiple structural characteristics determined by FRI were associated with abnormalities determined by CF-CT score. Air trapping and pulmonary blood distribution appeared to be the most clinically relevant FRI parameters for CF patients due to their associations with classical outcome measures. The FRI methodology could particularly be of interest for patients with mild lung disease, although this should be confirmed in future research.

**Supplementary Information:**

The online version contains supplementary material available at 10.1186/s12890-021-01622-3.

## Background

Cystic fibrosis (CF) is a genetic disease characterized by progressive lung disease starting in the first months of life. Although mutations of the *cystic fibrosis transmembrane conductance regulator* (*CFTR*) gene affect multiple organs, obstructive lung disease remains the major cause of morbidity and mortality in CF patients [1]. Computed tomography (CT) is universally accepted as the gold standard to evaluate structural abnormalities of the airways and lung parenchyma, including bronchiectasis, mucus plugging, bronchial wall thickening and air trapping [2]. Previous research has shown that structural changes are detected by CT imaging well before they are reflected in any classical lung function parameter, and that these findings have an important impact on clinical management [3, 4]. To allow structural abnormalities to serve as endpoints in clinical research, these markers would need to be quantifiable. Visual scoring methods (e.g. Bhalla [5], Brody [6], CF-CT [7], PRAGMA-CF [8]) have been frequently used over the past decades to measure disease progression and to evaluate treatment efficacy [9, 10]. Although multiple scoring systems have shown to be reproducible and correlate with other respiratory outcomes [10, 11], they have several limitations. Most importantly, these methods rely on subjective observations to produce quantitative measures, which inevitably leads to considerable intra- and interobserver variability. The reported intra-class correlation coefficients (ICCs) to measure inter-rater and intra-rater agreement range from 0.71 to 0.96 and from 0.76 to 0.98 for total scores, respectively [10]. In addition, most methods lack standardization, are time consuming and are relatively insensitive to early or small structural changes [9, 12]. To overcome many of the above-mentioned shortcomings, a smaller number of studies have described approaches for (semi-)automated quantification of low- or high attenuated areas and bronchial dilation [13–16]. This type of approach is focused on a specific CT feature and the choice depends on the research question to be addressed [17].

Our research project focuses on a novel technique in the field of CF research, namely Functional Respiratory Imaging (FRI). The semi-automated FRI technology distinguishes itself by providing a set of quantitative biomarkers analyzing structural as well as functional characteristics of the airways and lungs. These quantitative biomarkers allow for an in-depth description of respiratory health and post-treatment effects [18]. Previous studies have demonstrated the validity and responsiveness of FRI parameters in other obstructive airway diseases, including COPD and asthma [19–26]. FRI parameters, such as airway volume and airway resistance, have shown to be more sensitive to regional changes in the respiratory system compared to conventional pulmonary function outcomes [20, 23, 27]. Highly sensitive quantitative biomarkers could be of great benefit for CF research considering the increasing availability of highly effective CFTR modulator therapy and delayed disease progression. Since FRI studies including CF patients are scarce, the role of the acquired imaging parameters remains unclear in this population. Therefore, this study aims to explore the relation of FRI parameters to validated imaging parameters and classical respiratory outcomes.

## Methods

### Study population

In this cross-sectional study, chest CTs from participants of three other prospective clinical studies were collected for secondary analyses. In all three studies chest CTs were performed according to the same scanning protocol required for FRI analysis as outlined below, and similar eligibility criteria were applied. Results of these individual studies have been described elsewhere or will be published in the near future [28, 29]. All subjects were recruited at the Antwerp University Hospital between May 2017 and January 2020. Subjects were eligible for inclusion if they met the following criteria: documented diagnosis of CF, age > 5 years and clinically stable at inclusion. Patients with cognitive impairment were excluded. A summary of the study protocols of the three individual studies can be found in the supplementary material. Written informed consent was obtained from all enrolled subjects and their parents/guardians in case the subject was a minor. The study was approved by the local Ethics Committee of the Antwerp University Hospital.

### Anthropometrics, spirometry and 6-Minute Walk Test

Body length, body weight and BMI were assessed prior to other study assessments. Spirometry was performed using the Jaeger Masterscreen PFT (CareFusion, USA) or the Spirostik (Geratherm Respiratory, Germany) according to ERS standards [30]. Reference equations of the Global Lung function Initiative-2012 (GLI-2012) were used for predicted values. Exercise tolerance was evaluated by the 6-Minute Walk Test (6MWT) in accordance with the ATS/ERS guidelines [31]. Although the 6MWT is often referred to in a context of advanced lung disease, results of this test have shown to be reproducible and reliable in children with mild lung disease as well and they could be valuable to predict the risk for hospitalization [32–34]. SpO_2_ was measured with a pulse oximeter at the start of the test and every following minute during the 6MWT.

### CT imaging

Low-dose HRCT scans were taken at two breathing levels, Total Lung Capacity (TLC) and Functional Residual Capacity (FRC), monitored by a pneumotachograph. The scans were acquired with a 64-slice GE VCT LightSpeed scanner. A description of specific CT settings can be found in the supplementary material. The CF-CT scoring system, a validated upgraded version of the Brody II score, was used to quantify structural abnormalities on chest CT [6, 7]. The latter method was selected as it is one of the most frequently used visual scoring systems in current CF research and it offers an extensive training module to improve standardization. The images were reviewed by two independent observers blinded to subject ID and FRI outcomes, of whom one experienced chest radiologist and one certified researcher trained in CF-CT scoring. Following components were scored: bronchiectasis, bronchial wall thickening, mucus plugging, parenchymal abnormalities and air trapping. All scores were expressed as a percentage of the maximum score.

### Functional respiratory imaging

Prior to any analyses, FRI analysts were blinded to all corresponding patient characteristics. A patient-specific 3D-model of the lung lobes, airways and vasculature is reconstructed via automated segmentation using the medical imaging processing software package, Mimics (Materialise, Leuven, Belgium). The bronchial tree can be segmented down to airways with a diameter of ca. 1–2 mm. These models are used to determine following parameters: lobar volume, airway volume, airway wall volume and air trapping. After segmentation and postprocessing, the models are used for computational fluid dynamics (CFD) simulations by solving Reynolds-averaged Navier–Stokes equations, to calculate regional airway resistance. These CFD calculations adjust the outflow iteratively for each patient to match the internal flow rate distributions obtained from the segmentation of the CT scans. Airway resistance is subsequently defined as the total pressure drop over an airway, divided by the flow rate through that airway. Airway volume, airway wall volume and airway resistance are corrected for lobar volume to allow comparison between subjects. Pulmonary vasculature is segmented using algorithms based on shape analysis to recognize tubular structures. The cross-sectional area of each identified blood vessel is determined to compute pulmonary blood distribution. The proportion of pulmonary blood volume of vessels with a cross-sectional area smaller than < 5 mm^2^ (BV5%), vessels between 5 and 10 mm^2^ (BV5_10%) and vessels larger than 10 mm^2^ (BV10%) are compared. All parameters were calculated at TLC level, except for air trapping and lobar volume at FRC level. A more detailed description of the analysis can be found in the supplementary material and in previously published work [19, 35, 36].

### Statistical analyses

Statistical analyses were performed in R for statistical computing (version 4.0.3, R Core Team 2020, Austria). A pooled analysis from three prospective studies was conducted, which all performed a sample size calculation a priori depending on the respective research questions. Since air trapping has previously been described as one of the most clinically relevant imaging parameters in CF, we expected a minimum correlation of r = 0.70 between air trapping determined by FRI and the percentage predicted of forced expiratory volume in 1 s (ppFEV_1_). Therefore, a minimum sample size of 13 would be needed for our correlation analysis to reach a power of 80% with a significance level of 0.05. To examine differences between patient characteristics of the three individual studies, the Kruskal–Wallis test was computed. The distribution of the data was evaluated by QQ plots and the Shapiro–Wilk test. Normally distributed data are represented as mean ± standard deviation, and non-normal data as median [range]. The interobserver reliability of the CF-CT scoring was examined by Bland–Altman plots and intraclass correlation coefficients (ICCs) using a two-way mixed-effects model for average measures [37]. The association between FRI parameters and CF-CT scores per lung lobe was investigated by a repeated measures correlation [38]. In addition, linear mixed-effects models were computed with subject and lung lobe as random effects. Similar mixed effects models with only subject as a random effect were computed for the comparison between FRI parameters and spirometry or 6MWT outcomes. To simplify the interpretation of the relation between parameters, a Pearson or Spearman correlation was calculated of a selection of scans of only one scan per subject. A subgroup analysis was performed to differentiate between patients with mild lung disease (ppFEV_1_ ≥ 70%) and patients with moderate to severe lung disease (ppFEV_1_ < 70%). For all analyses a *p* value < 0.05 was considered statistically significant.

## Results

Thirty-nine chest CTs from 24 subjects were collected. Patient characteristics at the time of the first CT are shown in Table 1. Additional file 1: Table S1 shows the patient characteristics for each individual study population separately, but no significant differences were noted between these three studies. For the subgroup analysis, 18 chest CTs from 12 subjects were categorized as mild lung disease (ppFEV_1_ ≥ 70%), and 21 chest CTs from another 12 subjects as moderate to severe lung disease (ppFEV_1_ < 70%). All measurements were performed on the same day, except for the 6MWT in six patients as the 6MWT was not performed as part of the study protocol in this group. For three of those patients the results of the most recent 6MWT were retrospectively added with a time difference of respectively 1, 6 and 8 weeks. For the remaining three patients, the results of the 6MWT were not retained, since the last tests were performed more than 10 months earlier. A summary with the results of all parameters that were assessed is presented in the Additional file 1: Tables S2–S5.

**Table 1:** Patient characteristics (n = 24)

Sex (M/F)	16/8
Age (years)	24 ± 9
Body length (cm)	169 ± 14
Body weight (kg)	62.9 ± 14.9
BMI (kg/m^2^)	21.81 ± 3.73
BMI z-score ≤ 21 years, n = 10	− 0.22 [− 1.92; 1.37]
ppFEV_1_ (%)	71 ± 25

Data are presented as mean ± standard deviation or median (range)

The interobserver variability analysis of the two observers using the CF-CT scoring method showed moderate to excellent absolute agreement with ICCs between 0.46 (bronchial wall thickening) and 0.92 (bronchiectasis). The consistency between the observers was higher with ICCs between 0.67 (bronchial wall thickening) and 0.93 (bronchiectasis). Overall, the Bland–Altman plots showed an increase in differences between observers for higher mean scores. A detailed overview of all ICCs and Bland–Altman plots can be found in the Additional file 1: Table S6, Figures S1–S6.

### FRI versus CF-CT

**Fig. 1:**
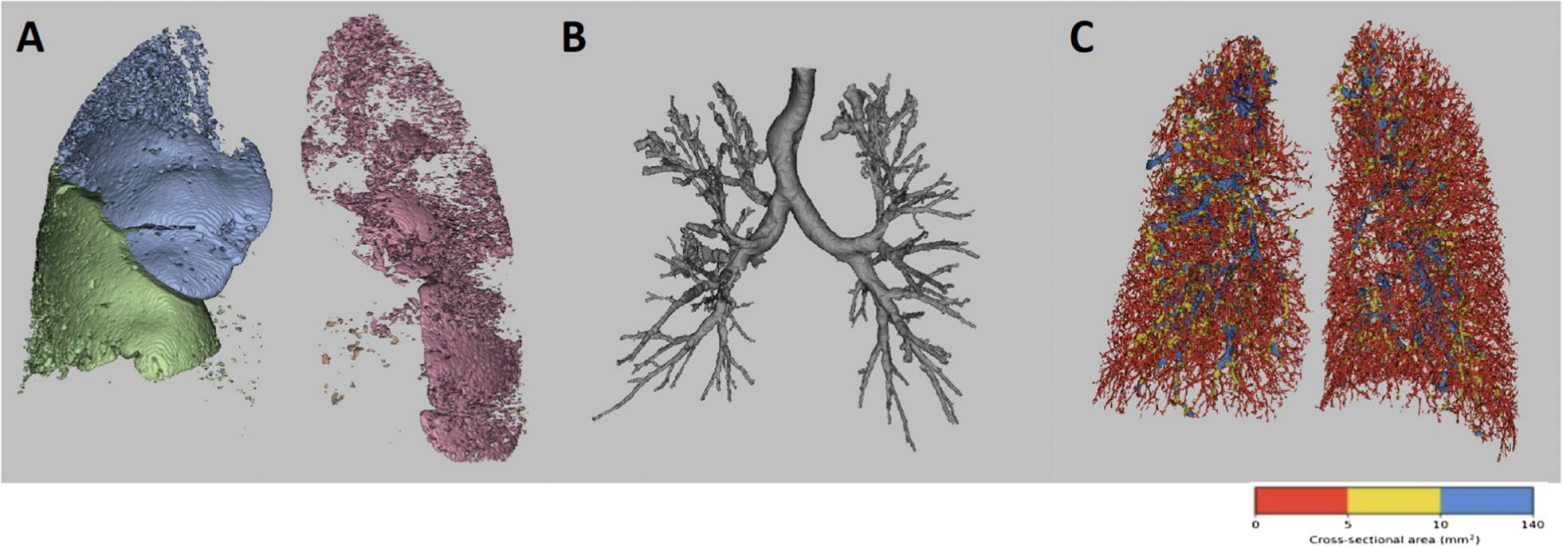
Illustration of FRI parameters: **A** air trapping, **B** airway volume at TLC, **C** pulmonary vessels with a cross-sectional area < 5 mm^2^, 5–10 mm^2^ and > 10 mm^2^, respectively coloured in red, yellow and blue

**Fig. 2:**
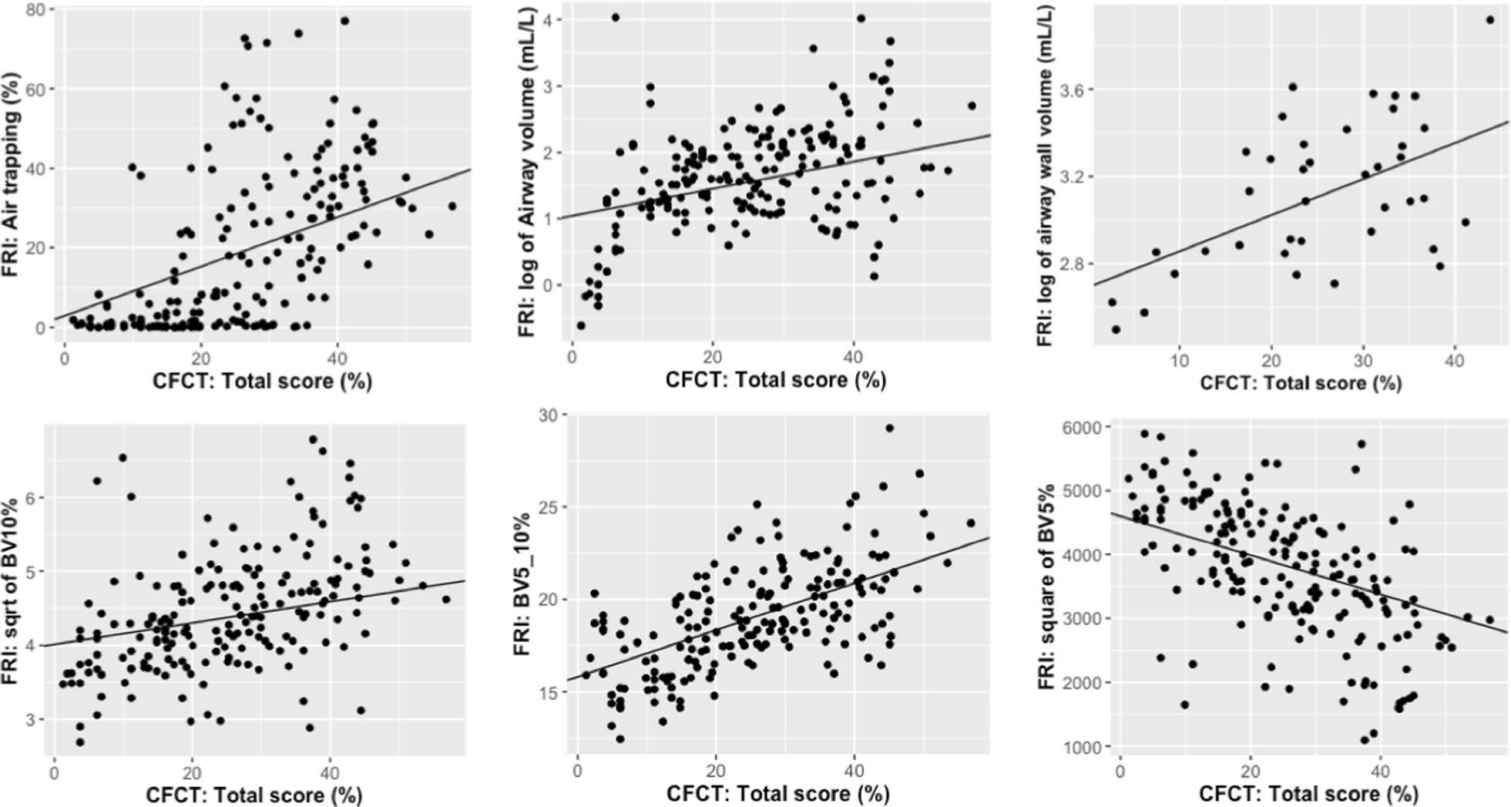
Association between FRI parameters and the CF-CT total score of all lobes with the regression lines of the linear mixed effects models

An illustration of the segmentation and visualization of several FRI parameters of interest are shown in Fig. 1. The 3D visualizations show the results of a male CF patient of 37 years old with a ppFEV_1_ of 72%. Results of the three FRI features were all close to the mean values of the study sample (air trapping: 16.04%, airway volume: 11.30 mL/L, BV10%: 18.87, BV5_10%: 17.87, and BV5%: 63.26%). The repeated measures correlation analysis showed low-to-moderate correlations between multiple FRI parameters and CF-CT scores. The total CF-CT score per lung lobe was related to air trapping (r = 0.43, *p* < 0.001), airway volume (r = 0.35, *p* < 0.001), airway wall volume (r = 0.30, *p* < 0.001) and airway resistance (r = − 0.15, *p* = 0.0495). Other relevant correlations were those between airway volume and % bronchiectasis (r = 0.40, *p* < 0.001), and between air trapping on FRI and % air trapping of the CF-CT score (r = 0.38, *p* < 0.001). The correlation between airway wall volume and % bronchial wall thickness was low, but still significant (r = 0.18, *p* = 0.02). Proportion of BV5_10% and BV10% were positively correlated with total CF-CT score (BV5_10%: r = 0.57, *p* < 0.001; BV10%: r = 0.16, *p* = 0.04) (Fig. 2). On the other hand, BV5%, representing the proportion blood vessels with a cross-sectional area smaller than 5 mm^2^, showed a negative correlation with total CF-CT score (r = − 0.34, *p* < 0.001). Also, a marked correlation was found between total pulmonary blood volume corrected for lobar volume and % mucus (r = 0.41, *p* < 0.001). An overview of all pairwise comparisons is shown in Table 2. When considering the results of the subgroup analysis to differentiate between mild and moderate to severe lung disease, a notable difference between the two groups became apparent. The correlations between the two image analyses of the scans from patients with mild lung disease were overall much higher compared to the results of the entire group and those of moderate to severe lung disease. Significant repeated measures correlation coefficients with the CF-CT total score from patients with mild lung disease were the following: air trapping (r = 0.49, *p* < 0.001), airway volume (r = 0.51, *p* < 0.001), airway wall volume (r = 0.66, *p* < 0.001), airway resistance (r = − 0.32, *p* = 0.005), BV5% (r = − 0.48, *p* < 0.001), BV5_10% (r = 0.72, *p* < 0.001) and BV10% (r = 0.25, *p* = 0.03). On the other hand, only airway volume (r = 0.30, *p* = 0.004), air trapping (r = 0.43, *p* < 0.001), BV5% (r = − 0.26, *p* = 0.01) and BV5_10% (r = 0.49, *p* < 0.001) correlated significantly with the CF-CT total score in patients with moderate to severe lung disease. Results of the subgroup analysis can be found in the Additional file 1: Tables S7–S8).

**Table 2:** Correlations FRI versus CF-CT (n = 39)

FRI	CF-CT scores (%)					
	BE	Mucus	BWT	Parenchyma	AT	Total
iVlobe at TLC (L)	NS	NS	NS	− 0.17*	NS	NS
Sqrt of iVlobe at FRC (L)	NS	NS	0.18*	NS	NS	NS
Log of siVaw at TLC (mL/L)	0.40***	NS	0.17*	0.26***	0.23**	0.35***
Log of siVaww at TLC (mL/L)	0.30***	0.16*	0.18*	0.36***	NS	0.30***
Cube root of siRaw at TLC (kPa * s/L)	NS	NS	NS	− 0.21**	− 0.23**	− 0.15*
AT at FRC (%)	0.37***	0.35***	0.29***	0.21**	0.38***	0.43***
Sqrt of TBV at TLC (mL/L)	NS	0.41***	0.16*	0.32***	NS	0.20*
Sqrt of BV10% at TLC (%)	NS	0.19*	NS	0.37***	NS	0.16*
BV5_10% at TLC (%)	0.55***	0.43***	0.50***	NS	0.41***	0.57***
Square of BV5% at TLC (%)	− 0.22**	− 0.32***	− 0.26***	− 0.40***	− 0.22**	− 0.34***

Repeated measures correlation coefficients, *p < 0.05; **p < 0.01; ***p < 0.001

*iVlobe* lobar volume, *siVaw* specific airway volume, *siVaww* specific airway wall volume, *siRaw* specific airway resistance, *AT* air trapping, *TBV* total pulmonary blood volume corrected for lobar volume, *BV10%* percentage of blood vessels larger than 10 mm^2^, *BV5_10%* percentage of blood vessels between 5 and 10 mm^2^, *BV5%* percentage of blood vessels smaller than 5 mm^2^, *BE* bronchiectasis, *BWT* bronchial wall thickening

### FRI versus classical clinical outcome measures

**Fig. 3:**
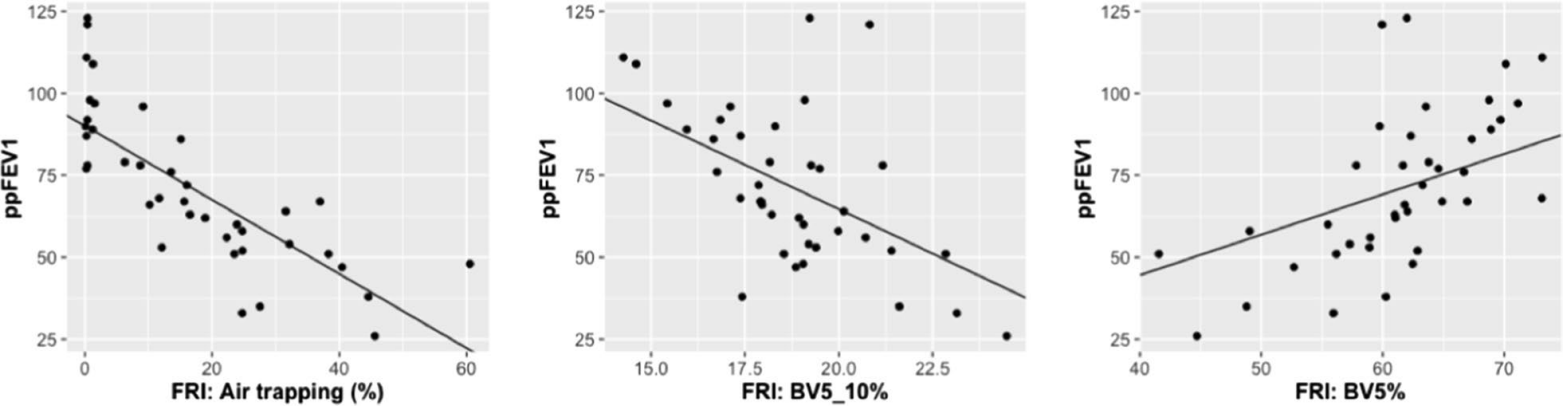
Association between FRI parameters and ppFEV_1_ with the regression lines of the linear mixed effects models

The regression analyses including all 39 scans showed a significant association between ppFEV_1_ and air trapping, lobar volume at FRC, airway resistance and all vascular parameters. When considering only one scan per subject, the correlation coefficients of ppFEV_1_ and the latter FRI parameters were the following: air trapping: r = − 0.85 (*p* < 0.001), lobar volume at FRC: r = − 0.72 (*p* < 0.001), BV10%: r = − 0.57 (*p* = 0.003), BV5_10%: r = − 0.61 (*p* = 0.002), and BV5%: *r* = 0.64 (*p* < 0.001) (Fig. 3). No significant correlation was found between ppFEV_1_ and airway resistance, despite the result of the regression model.

Of all FRI parameters, only air trapping was significantly associated with the distance covered during 6MWT (6MWD). On the other hand, SpO_2_ measured at the end of 6MWT was associated with air trapping, lobar volume at FRC, BV5_10% and BV5%. Regarding the CF-CT total score and subscores, all of them showed significant correlations with spirometry. Correlations with ppFEV_1_ ranged from r = − 0.61 to r = − 0.85. In addition, total CF-CT score correlated with 6MWD (r = − 0.53) and oxygen saturation at the end of 6MWT (r = − 0.73). Results of all spirometric parameters and 6MWT can be found in Tables 3 and 4.

**Table 3:** Correlations imaging versus spirometry (n = 24)

	Spirometry (% predicted)				
	FEV_1_	FVC	Square of PEF	Sqrt of MEF_25–75_	Log of MEF_25_
FRI					
iVlobe at TLC (L)	NS	NS	NS	NS	NS
iVlobe at FRC (L)	− 0.72***	− 0.43*	− 0.66***	− 0.77***	− 0.76***
Log of siVaw at TLC (mL/L)	NS	NS	NS	NS	NS
Log of siVaww at TLC (mL/L)	NS	− 0.44*	NS	NS	NS
Log of siRaw at TLC (kPa * s/L)	NS	NS	NS	NS	NS
AT at FRC (%)	− 0.85***	− 0.59**	− 0.76***	− 0.88***	− 0.84***
TBV at TLC (mL/L)	NS	NS	NS	NS	NS
BV10% at TLC (%)	− 0.57**	− 0.51*	− 0.59**	− 0.58**	− 0.45*
BV5_10% at TLC (%)	− 0.61**	− 0.59**	− 0.49*	− 0.54**	− 0.55**
Square of BV5% at TLC (%)	0.64***	0.57**	0.62**	0.61**	0.52**
CF-CT					
Total score (%)	− 0.85***	− 0.73***	− 0.65***	− 0.80***	− 0.81***
Bronchiectasis (%)	− 0.77***	− 0.70***	− 0.51*	− 0.73***	− 0.73***
Mucus plugging (%)	− 0.80***	− 0.68***	− 0.69***	− 0.75***	− 0.77***
BWT (%)	− 0.61**	− 0.58**	NS	− 0.50*	− 0.58**
Parenchyma (%)	− 0.73***	− 0.77***	− 0.48*	− 0.66***	− 0.66***
AT (%)	− 0.69***	− 0.54**	− 0.61**	− 0.65***	− 0.66***

FRI: Pearson correlation coefficients, only iVlobe at FRC and AT at FRC were calculated using Spearman. CF-CT: Spearman correlation coefficients. *p < 0.05; **p < 0.01; ***p < 0.001

*iVlobe* lobar volume, *siVaw* specific airway volume, *siVaww* specific airway wall volume, *siRaw* specific airway resistance, *AT* air trapping, *TBV* total pulmonary blood volume corrected for lobar volume, *BV10%* percentage of blood vessels larger than 10 mm^2^, *BV5_10%* percentage of blood vessels between 5 and 10 mm^2^, *BV5%* percentage of blood vessels smaller than 5 mm^2^, *BWT* bronchial wall thickening

**Table 4:** Correlations FRI versus 6MWT (n = 21)

	6MWT		
	6MWD (m)	SpO_2_ start (%)	SpO_2_ end (%)
FRI			
iVlobe at TLC (L)	NS	NS	NS
iVlobe at FRC (L)	NS	NS	− 0.69***
Log of siVaw at TLC (mL/L)	NS	NS	NS
Log of siVaww at TLC (mL/L)	NS	NS	NS
Log of siRaw at TLC (kPa*s/L)	NS	NS	NS
AT at FRC (%)	− 0.85***	NS	− 0.72***
TBV at TLC (mL/L)	NS	NS	NS
BV10% at TLC (%)	NS	NS	− 0.59**
BV5_10% at TLC (%)	NS	NS	− 0.60**
Square of BV5% at TLC (%)	NS	NS	0.53*
CF-CT			
Total score	− 0.53*	NS	− 0.73***
Bronchiectasis	NS	NS	− 0.72***
Mucus plugging	NS	NS	− 0.66**
BWT	NS	NS	NS
Parenchyma	− 0.45*	NS	NS
AT	NS	NS	− 0.71***

FRI: Spearman correlation coefficients, only 6MWD was calculated using Pearson. CF-CT: Spearman correlation coefficients. *p < 0.05; **p < 0.01; ***p < 0.001

*iVlobe* lobar volume, *siVaw* specific airway volume, *siVaww* specific airway wall volume, *siRaw* specific airway resistance, *AT* air trapping, *TBV* total pulmonary blood volume corrected for lobar volume, *BV10%* percentage of blood vessels larger than 10 mm^2^, *BV5_10%* percentage of blood vessels between 5 and 10 mm^2^, *BV5%* percentage of blood vessels smaller than 5 mm^2^, *BWT* bronchial wall thickening

In contrast to the results of the entire group, the subgroup analysis added little information. Since only 12 subjects were considered in both the group with mild lung disease and with moderate to severe lung disease, hardly any of the regression models or correlation analyses showed significant results. Therefore, no relevant comparison between the two levels of airway obstruction could be made.

## Discussion

FRI analysis comprises several computational algorithms to quantitatively analyze structural and functional components of the respiratory system. Regarding the functional variables, only the calculation of regional airway resistance was considered relevant for further analysis. Additional functional information regarding changes in regional flow distribution or aerosol deposition were not applicable for this study design. Structural variables and regional airway resistance determined by FRI analyses were compared to validated imaging parameters and classical respiratory outcomes in this cross-sectional study. Various correlations were found between structural FRI parameters and CF-CT scores. All parameters were significantly correlated to the total CF-CT score, except for lobar volume. When considering the relation between FRI parameters and similar CF-CT subscores, significant correlations were found between parameters related to airway volume, air trapping and airway wall thickening. The strength of the correlations was overall interpreted as low-to-moderate, which was expected since the definitions of the parameters and the analyses differ considerably.

FRI parameters related to hyperinflation, i.e. air trapping and lobar volume at FRC, and pulmonary blood distribution showed significant associations with all spirometric parameters and oxygen saturation at the end of 6MWT. In addition, air trapping was the only parameter related to the distance covered during 6MWT. It should be noted that in this heterogenous group of patients the distance in meters was not corrected for age, sex or body length, since no appropriate reference equations were available. Air trapping, as an indirect measure of small airway obstruction, has been studied extensively in previous CF research using expiratory chest CT [9, 15, 39]. Since the extent of trapped air is partially reversible in CF, this structural feature is considered a meaningful outcome to evaluate post-treatment effects in clinical practice as well as for clinical trials [16, 40]. In our study, FRI detected < 1% air trapping in 9/39 scans. Since the presence of trapped air has been described to be an early marker of CF lung disease [41], and seven of these nine scans scored > 5% on CF-CT air trapping and CF-CT total score, FRI probably underestimated the extent of this structural abnormality. One explanation could be that the expiratory scans were taken at FRC level instead of residual volume (RV). Regions of trapped air would have been more pronounced at RV, but the flow simulation models of the FRI methodology have been developed using FRC as a more functional breathing level. By avoiding a forced expiratory maneuver, such as a breath-hold at RV, this methodology is more accessible for a wide range of respiratory patients. However, when comparing our results to previous CF research involving expiratory scans at RV, this discrepancy in lobar volume needs to be taken into account.

Besides air trapping, the distribution of pulmonary blood volume correlated with multiple respiratory outcomes. Pulmonary vascular disease as a result of hypoxia-induced vasoconstriction is recognized as a predictor of morbidity and mortality in CF, but investigation of pulmonary hypertension has been primarily limited to those with advanced lung disease [42]. The results of our study suggest a redistribution of blood related to ventilation defects caused by for example mucus plugging. An average increase in pulmonary vascular resistance in patients with more severe lung disease is indicated by a relative shift of blood volume from smaller to larger blood vessels. These results, however, should be interpreted with caution, since the automated segmentation of the pulmonary vasculature was probably biased by the presence of mucus plugs and other opacities. Mucus plugs can present as tubular structures with a similar density compared to blood vessels, which could have led to false positives [35]. This would explain the positive correlation between the total blood volume and the CF-CT score for mucus plugs and parenchymal abnormalities. To what extent the mucus plugs and areas of consolidation biased the vascular parameters should be verified by more in-depth analyses. For that reason, a follow-up study will be conducted combining chest CT with intravenous contrast to objectively quantify any misclassifications and to adapt the algorithms accordingly if needed.

Interestingly, hardly any clinically relevant correlations were found for airway resistance determined by FRI. Contrary to airway resistance measured by body plethysmography, airway resistance determined by FRI is calculated locally in the airways and is thereby related to airway volume. The airway volume of patients in CF will overall be greater than in healthy controls due to the presence of bronchiectasis, which is confirmed by the positive correlation between airway volume determined by FRI and the CF-CT bronchiectasis score. In addition, this also explains the (small) negative correlations between FRI airway resistance and CF-CT scores. As FRI only considers the volume of lumen area to calculate airway volume, mucus retention against the airway walls will reduce airway volume and increase airway resistance locally. Therefore, the latter parameters are suitable to reflect acute effects of mucus shifting and clearance efficacy in CF as demonstrated in a previous study by Leemans et al. [28]. Although airway volume and airway resistance by FRI have been used as primary outcomes in multiple studies including patients with COPD and asthma [20, 23–26], their value in CF research in long-term trials still needs to be evaluated. While these characteristics in COPD and asthma are predominantly influenced by airway narrowing, a more heterogeneous presentation arises in CF resulting from both airway narrowing (e.g. mucus retention, airway inflammation) and airway dilation (bronchiectasis), which complicates the interpretation. The heterogeneity of regional airway volume and airway resistance in this population probably explains the lack of correlation with classical respiratory outcomes in our study that reflect the respiratory system as a single unit.

A subanalysis was performed to differentiate between patients with mild and moderate to severe lung disease. The correlation coefficients between the CF-CT scores and FRI parameters were considerably higher in the subgroup with mild lung disease. One of the notable results was the increased correlation between airway wall volume determined by FRI and CF-CT total score and bronchial wall thickening. As bronchial wall thickening is a well-known early marker for progressive lung disease, this could be an appropriate outcome in future CF research. Unfortunately, the subgroups were too small to relate imaging parameters to other clinical outcomes. In contrast to the statistical analysis between FRI and CF-CT, only one total value per imaging feature (e.g. total air trapping) could be related to a clinical outcome. As FRI and CF-CT are compared on a lobar level, less participants were required to achieve sufficient power.

Although the correlations of the FRI parameters in the general study group were comparable or lower than those between the CF-CT scores and classical outcomes, FRI has several advantages over conventional pulmonary function tests, the 6MWT and visual scoring systems: (1) as mentioned earlier FRI is able to reflect the heterogeneity of the respiratory disease by providing regional information; (2) FRI parameters provide objective quantitative information; (3) several FRI parameters have shown to be more sensitive to detect small changes than conventional endpoints in clinical trials including other pathologies. Preliminary results of a study by our research group including CF patients receiving CFTR modulator therapy (lumacaftor/ivacaftor) are in accordance with the latter statement [29], but larger clinical trials investigating CF interventions are needed to confirm. Despite the potential benefits, our study and the FRI analysis are subject to several limitations. Some have been mentioned previously, such as the potential impact of mucus and opacities on the calculation of the vascular parameters and the influence of the breathing level on air trapping. In addition, it must be acknowledged that both air trapping described by FRI and by CF-CT represent hypoattenuated regions on expiratory CT, and therefore represent the extent of trapped air as well as hypoperfusion [9]. Second, no healthy controls were included in the study nor appropriate reference values for FRI parameters were available to interpret values adjusted for age, body height and sex. However, most parameters were corrected for lobar volume to enable comparison between subjects. Third, no information is available on how changes in FRI parameters are related to changes in conventional outcome measures as only a cross-sectional evaluation was made. Lastly, a critical remark about the radiation exposure associated with CT imaging. To date, low-dose protocols minimize the radiation burden, but frequent CT scanning, especially in the pediatric population, must be prevented [43].

Future research is needed to focus on the long-term changes of FRI parameters related to other clinical endpoints. Our subgroup analysis suggests that the FRI methodology as presented in this study could be especially of interest to patients with mild lung disease. Bearing in mind the delayed progression of CF lung disease and upcoming highly effective CFTR modulator therapy as mentioned in the introduction, sensitive outcomes to detect early and/or small changes are most needed. However, the number of patients in our study with varying levels of airway obstruction were too low to draw any meaningful conclusions. Additional studies are needed to investigate the latter finding. Also, the addition of functional information as one of the key benefits of the technology should be further verified in this population, since regional airway resistance the only functional characteristic considered in this study design. Lastly, additional endpoints that have not been included in this study, such as time to pulmonary exacerbation and patient-reported outcomes, will provide more insight into the role of FRI in CF research.

## Conclusion

To conclude, FRI comprises a set of biomarkers reflecting structural as well as functional characteristics of the respiratory system on a lobar level. This study investigated the relationship of multiple FRI parameters with validated imaging parameters and classical respiratory outcomes in patients with CF. Despite differences in definition and in image analysis, all FRI parameters showed significant correlations with structural abnormalities determined by the CF-CT scoring system. When considering the association with spirometry and the 6MWT, air trapping and pulmonary blood distribution appeared to be most relevant. Although one of the advantages of FRI over conventional chest CT is the addition of functional information, this was not reflected in the study results. The only functional component included was regional airway resistance, which did not show any clinically relevant correlations with classical outcomes. Nevertheless, the set of structural components of FRI providing quantitative, objective and regional information have the potential to complement results derived from conventional outcome measures in future CF research as an alternative to visual CT scores. Longitudinal studies will be needed to examine the value of specific FRI parameters to predict future disease progression in terms of lung function, exacerbation rate, quality of life, etc. Lastly, future studies should verify the opportunities of the FRI methodology in CF patients with mild lung disease.

## Supplementary Information

**Additional file 1**. Supplementary material.

## Data Availability

All data are summarized within the article and the supplementary material (Tables S2-5). Individual patient data are not publicly available, but they are available from the corresponding author upon reasonable request.

## Abbreviations

6MWD: 6-Minute walk distance
6MWT: 6-Minute Walk Test
AT: Air trapping
BE: Bronchiectasis
BMI: Body mass index
BV10%: Proportion of pulmonary blood in vessels with a cross-sectional area > 10 mm^2^
BV5_10%: Proportion of pulmonary blood in vessels with a cross-sectional area 5–10 mm^2^
BV5%: Proportion of pulmonary blood in vessels with a cross-sectional area < 5 mm^2^
BWT: Bronchial wall thickening
CF: Cystic fibrosis
CF-CT: Cystic fibrosis-computed tomography scoring system
CFD: Computational fluid dynamics
CFTR: Cystic fibrosis transmembrane conductance regulator
COPD: Chronic obstructive pulmonary disease
FRC: Functional residual capacity
FRI: Functional respiratory imaging
FVC: Forced vital capacity
HRCT: High resolution computed tomography
ICC: Intra-class correlation coefficient
iVlobe: Lobar volume
MEF_25-75_: Mean expiratory flow between 25 and 75% of FVC
MEF_25_: Maximal expiratory flow where 25% of FVC remains to be expired
PEF: Peak expiratory flow
ppFEV_1_: Percentage predicted of FEV1
siRaw: Specific airway resistance
siVaw: Specific airway volume
siVaww: Specific airway wall volume
SpO_2_: Saturation of peripheral oxygen
TBV: Total pulmonary blood volume
TLC: Total lung capacity
