# Supplementary material for: Functional respiratory imaging in relation to classical outcome measures in cystic fibrosis: a cross-sectional study
Source: BMC Pulm Med. 2021 Aug 4;21:256. doi: 10.1186/s12890-021-01622-3 (PMC8336350; doi:10.1186/s12890-021-01622-3)
Supplement: Supplementary file 1 — Additional file 1. Supplementary material. [file 12890_2021_1622_MOESM1_ESM.docx]

**Supplementary material**

1. **Supplemental methods**
   1. Study protocols

In this cross-sectional study, chest CTs from participants of three other prospective clinical studies were collected for secondary analyses. A summary of these three study protocols is provided.

- Study 1: This randomized, open label, crossover study by Leemans et al was already published in 2020 in Pediatric Pulmonology [1]. Their primary objective was to compare the short-term effects of a new high frequency chest wall oscillation (HFCWO) device (The Monarch Airway Clearance System) to a standard HFCWO device (The Vest Airway Clearance System) on mucus clearance in patients with cystic fibrosis (CF). The primary outcome was expectorated sputum during and immediately after one session of each HFCWO device. The secondary objective was to gain more insight into the working mechanism of the actions of the new HFCWO device using FRI. Chest CT scans were taken according to the FRI protocol before and after the treatment session of the new device only. Other secondary outcomes assessed before and after HFCWO treatment included: spirometry and Brody CT scores. Inclusion criteria were the following: subjects needed to be clinically stable during 4 weeks prior to the study and subjects had FEV_1_ %predicted between 30 and 90%. Patients were excluded if there was an anticipated hospitalization within 3 weeks of the study, if they had a history of pneumothorax or haemoptysis requiring embolization within 6 and 12 months (respectively) before the first study visit, and if the participants were unwilling or unable to perform HFCWO treatment. For our study, 6 baseline chest CTs were included for further analysis.
- Study 2: The second study was an open label, single arm study to assess the effects of 12 weeks of lumacaftor/ivacaftor treatment in CF patients homozygous for *F508del*. The study protocol is registered at the EU clinical trials register (EudraCT 2018-001573-24) and preliminary results can be found in the form of an abstract published at the ATS 2020 International Conference [2]. The study aimed to offer additional and novel information about the mode of action of lumacaftor/ivacaftor by assessing lung structure and function across multiple FRI parameters. Participants aged 12 years or older were eligible for inclusion if they had a confirmed diagnosis of CF homozygous for the *F508del* mutation, a ppFEV_1_ ≥ 50%, and if they were clinically stable during the 4 weeks prior to the study. Participants visited the hospital every four weeks during the 12-week period at which following study assessments were carried out: physical examination and spirometry. Other assessments were only performed at baseline and at 12 weeks: CT chest imaging, nitrogen multiple breath washout (N_2_MBW) test, Six Minute Walk Test (6MWT), sweat chloride test and the Cystic Fibrosis Questionnaire – Revised (CFQ-R). For our study, both scans at baseline and at week 12 of the 12 participants were included.
- Study 3: The third study was a cross-sectional, observational study to compare digitally recorded lung sounds to FRI parameters, CF-CT scores and classical respiratory outcomes. Results of this study have not yet been published. Study assessments were performed during the annual review of patients with CF, including chest CT imaging, spirometry, body plethysmography, 6MWT and digital lung auscultation. Subjects were eligible for inclusion if they met the following criteria: documented diagnosis of CF, age > 5 years and clinically stable at inclusion. Exclusion criteria were cognitive impairment and severe psychiatric illness. Nine participants completed all study assessments and were included in our study for secondary analyses.

It is important to emphasize that all chest CTs and subsequent FRI analyses were performed according to the same protocol as outlined below.

- 1. CT settings

Chest CTs were acquired by a GE VCT LightSpeed scanner using the following scanning protocol:

- rotation time: 0.6 sec
- detector coverage: 40 mm
- helical thickness: 0.625 mm
- pitch and speed: 1.375:1 and 55 mm/s
- tube voltage: 100 kV
- tube current: variable between 10 and 200 mAs
- noise index: 45
  1. Functional Respiratory Imaging

The reconstruction of the lung lobes and bronchial tree are performed in the commercially available software package, Mimics (Materialise NV, Belgium). Images are reconstructed at 0.3 mm interval using a lung filter. The resulting data set has 700-1200 images with a pixel size of 0.4-0.65 mm^2^. Lung volumes are segmented using a HU threshold of [-1024; -400]. The fissure lines are manually identified to separate the lung lobes. The bronchial tree, i.e. intraluminal air, is automatically segmented up to a point where no distinction can be made between intraluminal and alveolar air, which is down to a level of airways with a diameter of 1-2 mm. A typical airway model includes 5-10 generations, depending mainly on the disease state of the individual patient. More generations will be identified in patients with cystic fibrosis with extensive bronchiectasis. Air trapping can be determined through segmentation based on Hounsfield unit (HU) thresholds of [-1024; -850] performed on the FRC scan.

Functionality is added to the static segmented images by applying computational fluid dynamics (CFD) methods to characterize airway resistance. The airway models are converted into a computational grid in order to solve the Navier-Stokes flow equations numerically, using commercial software packages (Ansys Inc., Canonsburg, PA, USA).  During the CFD calculations, the outflow to each lobe is adjusted iteratively for each patient to match the internal flow rate distributions obtained from the segmentation of the CT scans. Therefore, the airflow distribution in the CFD calculation reflects the airflow distribution as derived from the expansion of the lung lobes from FRC to TLC. Subsequently, airway resistance is defined as the total pressure drop over an airway divided by the flow rate through that airway.

A reconstruction of the pulmonary vasculature is made by a segmentation algorithm that performs an eigenvalue analysis of the Hessian matrix to recognize tubular structures. The probability of each voxel belonging to a tubular structure is returned based on shape analysis [3]. To limit vessels, HU thresholds are used based on vessel size and defined by an automated adaptive iterative threshold method. The cross-sectional area of each identified blood vessel is determined to compute pulmonary blood volume. This cross-sectional area is used to distinguish between smaller and larger blood vessels, namely pulmonary blood volume contained in vessels < 5 mm^2^, vessels between 5 and 10 mm^2^ and vessels > 10 mm^2^ (BV5, BV5_10 and BV10, respectively) [4].

1. **Supplemental results**
2. Patient characteristics of the three individual studies

| **Table S1. Patient characteristics** | | | | |
| --- | --- | --- | --- | --- |
|  | Study 1 (n = 6) | Study 2 (n = 12) | Study 3 (n = 9) | *P value* |
| Sex (M/F) | 2/4 | 1/11 | 4/5 |  |
| Age (y) | 25 [21; 36] | 24 [17; 46] | 22 [8; 40] | *0.43* |
| Body length (cm) | 168 [154; 186] | 174 [160; 187] | 170 [127; 180] | *0.27* |
| Body weight (kg) | 53.5 [49; 71] | 68 [47; 86] | 66 [25; 88] | *0.26* |
| BMI (kg/m^2^) | 20.17 [17.16; 22.92] | 23.49 [16.26; 26.95] | 21.89 [14.29; 30.09] | *0.34* |
| ppFEV_1_ (%) | 58 [38; 68] | 67.5 [47; 121] | 89 [26; 98] | *0.20* |
| Data are presented as mean ± standard deviation or median [range]. Kruskal-Wallis test was computed to examine differences between groups. | | | | |

1. Summary of data

| **Table S2. FRI** (total all lobes, n = 39) | |
| --- | --- |
| iVlobe at FRC (L) | 3.15 ±1.04 |
| iVlobe at TLC (L) | 5.57 ±1.37 |
| siVaw at TC (mL/L) | 11.37 ±4.23 |
| siVaww at TLC (mL/L) | 23.97 ±8.21 |
| siRaw at TLC (kPa*s) | 0.177 ±0.097 |
| AT at FRC (%) | 16.99 ±15.65 |
| BV10% at TLC (%) | 19.93 ±5.64 |
| BV5_10% at TLC (%) | 18.78 ±2.20 |
| BV5% at TLC (%) | 61.29 ±7.18 |
| TBV at TLC (mL) | 269.24 ±58.46 |
| Abbreviations: iVlobe, lobar volume; siVaw, specific airway volume; siVaww, specific airway wall volume; siRaw, specific airway resistance; AT, air trapping; TBV, total pulmonary blood volume corrected for lobar volume; BV10%, percentage of blood vessels larger than 10 mm^2^; BV5_10%, percentage of blood vessels between 5 and 10 mm^2^; BV5%, percentage of blood vessels smaller than 5 mm^2^ | |

| **Table S3. CFCT** (total all lobes, n = 39) | |
| --- | --- |
| Bronchiectasis (%) | 27.08 [0; 67.36] |
| Mucus plugging (%) | 30.56 [0; 62.50] |
| Bronchial wall thickness (%) | 32.18 [9.26; 49.07] |
| Parenchyma (%) | 8.33 [0; 25.93] |
| Air trapping (%) | 33.33 [0; 75.00] |
| Total score (%) | 24.18 [2.68; 43.83] |

| **Table S4. Spirometry** (n = 39) | |
| --- | --- |
| FEV_1_ (% pred) | 71.23 ±23.85 |
| FVC (% pred) | 85.62 ±14.58 |
| PEF (% pred) | 86.08 ±21.47 |
| MEF_25-75_ (% pred) | 53.68 ±40.30 |
| MEF_25_ (% pred) | 54.13 ±59.08 |

| **Table S5. 6MWT** (n = 33) | |
| --- | --- |
| Distance (m) | 649.30 ±97.00 |
| SpO_2_ at baseline (%) | 97 [92; 99] |
| SpO_2_ end exercise (%) | 94 [86; 98] |

1. CF-CT: interobserver variability analysis

| **Table S6.** Interobserver variability analysis | | | | |
| --- | --- | --- | --- | --- |
|  | **Absolute agreement** | | **Consistency** | |
|  | ICC | P value | ICC | P value |
| Total score | 0.78 [-0.06; 0.93] | < 0.001 | 0.90 [0.80; 0.95] | < 0.001 |
| Bronchiectasis | 0.92 [0.82; 0.96] | < 0.001 | 0.93 [0.86; 0.96] | < 0.001 |
| Mucus plugging | 0.64 [-0.20; 0.87] | < 0.001 | 0.83 [0.67; 0.91] | < 0.001 |
| Bronchial wall thickness | 0.46 [-0.20; 0.76] | < 0.001 | 0.67 [0.38; 0.83] | < 0.001 |
| Parenchyma | 0.76 [-0.15; 0.92] | < 0.001 | 0.90 [0.80; 0.95] | < 0.001 |
| Air trapping | 0.82 [0.66; 0.91] | < 0.001 | 0.82 [0.66; 0.91] | < 0.001 |

**Figures S1-6.** Bland-Altman plots to analyse the agreement between the two observers, EL and AS.


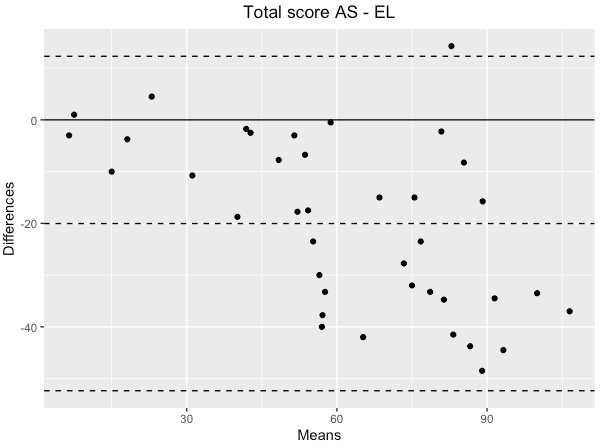

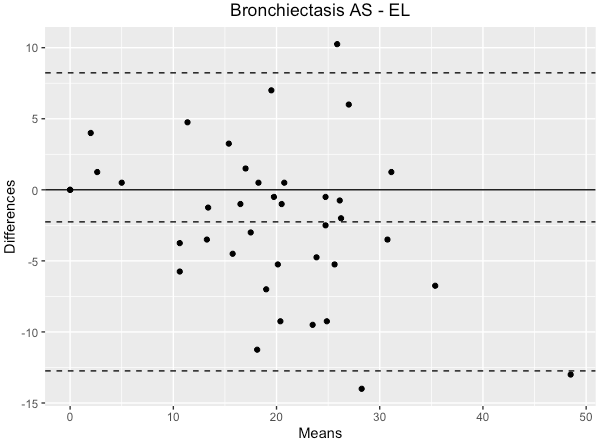


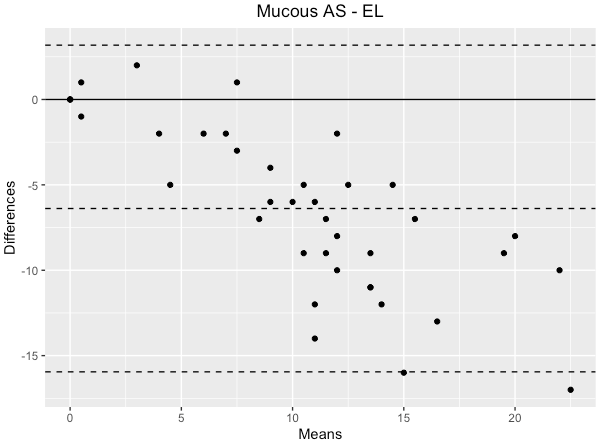

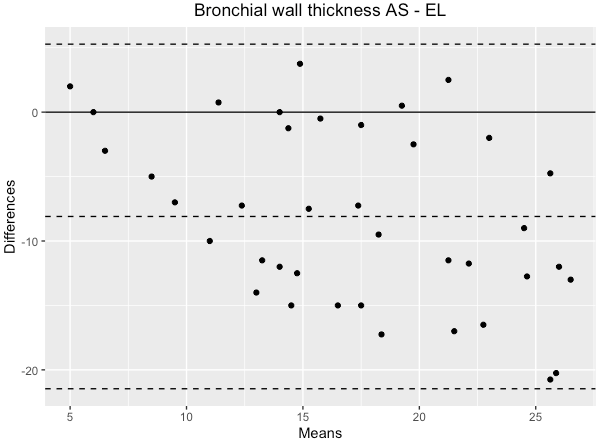


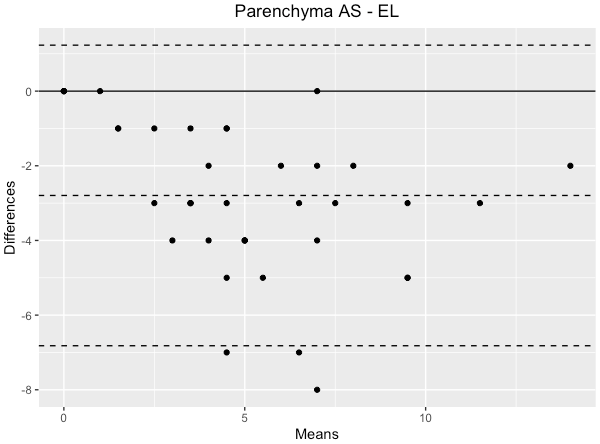

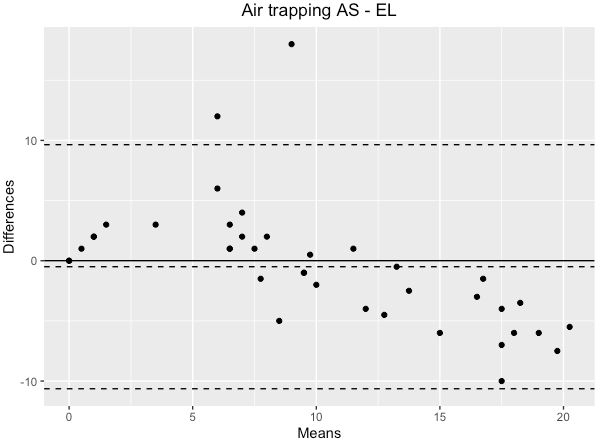


1. Subgroup analysis mild vs. moderate to severe lung disease

| **Table S7. Correlations FRI vs. CF-CT in patients with mild lung disease (n = 18).** | | | | | | | | | |
| --- | --- | --- | --- | --- | --- | --- | --- | --- | --- |
|  | |  | **CF-CT scores (%)** | | | | | | |
| **FRI** | *Transform* |  | BE | Mucus | BWT | Parenchyma | AT | Total score |  |
| iVlobe at TLC (L) | *-* |  | NS | NS | NS | NS | NS | NS |  |
| iVlobe at FRC (L) | *sqrt* |  | NS | 0.26* | NS | NS | NS | NS |  |
| siVaw at TLC (mL/L) | *sqrt* |  | 0.56*** | 0.32** | 0.42*** | 0.30** | 0.36** | 0.51*** |  |
| siVaww at TLC (mL/L) | *log* |  | 0.61*** | 0.48*** | 0.59*** | 0.36** | 0.53*** | 0.66*** |  |
| siRaw at TLC (kPa*s/L) | *log* |  | -0.29* | NS | -0.29* | -0.23* | -0.31** | -0.32** |  |
| AT at FRC (%) | *-* |  | 0.41*** | 0.32** | 0.34** | 0.33** | 0.59*** | 0.49*** |  |
| TBV at TLC (mL/L) | *log* |  | 0.26* | 0.42*** | 0.29* | 0.34** | 0.56*** | 0.43*** |  |
| BV10% at TLC (%) | *sqrt* |  | NS | NS | NS | 0.29* | 0.35** | 0.25* |  |
| BV5_10% at TLC (%) | *-* |  | 0.71*** | 0.48*** | 0.58*** | 0.40*** | 0.60*** | 0.72*** |  |
| BV5% at TLC (%) | *square* |  | -0.41*** | -0.34** | -0.32** | -0.39*** | -0.52*** | -0.48*** |  |
| Repeated measures correlation coefficients, *p < 0.05, ** p <0.01, *** p < 0.001. Abbreviations: BE, bronchiectasis; BWT, bronchial wall thickness; AT, air trapping; iVlobe, lobar volume; siVaw, specific airway volume; siVaww, specific airway wall volume; siRaw, specific airway resistance; TBV, total pulmonary blood volume corrected for lobar volume; BV10%, percentage of blood vessels larger than 10 mm^2^; BV5_10%, percentage of blood vessels between 5 and 10 mm^2^; BV5%, percentage of blood vessels smaller than 5 mm^2^. | | | | | | | | | |

| **Table S8. Correlations FRI vs. CF-CT in patients with moderate to severe lung disease (n = 21).** | | | | | | | | |
| --- | --- | --- | --- | --- | --- | --- | --- | --- |
|  | |  | **CF-CT scores (%)** | | | | | |
| **FRI** | *Transform* |  | BE | Mucus | BWT | Parenchyma | AT | Total score |
| iVlobe at TLC (L) | *-* |  | NS | NS | NS | NS | NS | NS |
| iVlobe at FRC (L) | *sqrt* |  | NS | NS | 0.24* | NS | NS | NS |
| siVaw at TLC (mL/L) | *log* |  | 0.37*** | NS | NS | 0.23* | NS | 0.30** |
| siVaww at TLC (mL/L) | *log* |  | NS | NS | NS | 0.33** | NS | NS |
| siRaw at TLC (kPa*s/L) | *cube root* |  | NS | NS | NS | NS | NS | NS |
| AT at FRC (%) | *-* |  | 0.36*** | 0.36*** | 0.29** | NS | 0.32** | 0.43*** |
| TBV at TLC (mL/L) | *log* |  | NS | 0.39*** | NS | 0.30** | NS | NS |
| BV10% at TLC (%) | *sqrt* |  | NS | NS | NS | 0.38*** | NS | NS |
| BV5_10% at TLC (%) | *-* |  | 0.47*** | 0.40*** | 0.45*** | NS | 0.31** | 0.49*** |
| BV5% at TLC (%) | *square* |  | NS | -0.32** | -0.25* | -0.37*** | NS | -0.26* |
| Repeated measures correlation coefficients, *p < 0.05, ** p <0.01, *** p < 0.001. Abbreviations: BE, bronchiectasis; BWT, bronchial wall thickness; AT, air trapping; iVlobe, lobar volume; siVaw, specific airway volume; siVaww, specific airway wall volume; siRaw, specific airway resistance; TBV, total pulmonary blood volume corrected for lobar volume; BV10%, percentage of blood vessels larger than 10 mm^2^; BV5_10%, percentage of blood vessels between 5 and 10 mm^2^; BV5%, percentage of blood vessels smaller than 5 mm^2^. | | | | | | | | |

**References**

[1] Leemans G, Belmans D, van Holsbeke C, Becker B, Vissers D, Ides K, et al. The effectiveness of a mobile high‐frequency chest wall oscillation (HFCWO) device for airway clearance. Pediatric Pulmonology 2020;55:1984–92. https://doi.org/10.1002/ppul.24784.

[2] Lauwers E, Belmans D, Mignot B, Ides K, van Hoorenbeeck K, van Holsbeke C, et al. Functional Respiratory Imaging to Assess the Short-Term Effects of Orkambi (Lumacaftor/Ivacaftor) on Lung Function in Patients with Cystic Fibrosis Homozygous for F508del. ATS 2020 International Conference American Thoracic Society International Conference Meetings Abstracts, American Thoracic Society; 2020, p. A7910–A7910. https://doi.org/10.1164/ajrccm-conference.2020.201.1_meetingabstracts.a7910.

[3] Yang J, Ma S, Sun Q, Tan W, Xu M, Chen N, et al. Improved Hessian multiscale enhancement filter. Bio-Medical Materials and Engineering, vol. 24, IOS Press; 2014, p. 3267–75. https://doi.org/10.3233/BME-141149.

[4] Lins M, Vandevenne J, Thillai M, Lavon BR, Lanclus M, Bonte S, et al. Assessment of Small Pulmonary Blood Vessels in COVID-19 Patients Using HRCT. Academic Radiology 2020;27:1449–55. https://doi.org/10.1016/j.acra.2020.07.019.
